# Strain-Specific Benefits of *Bacillus* on Growth, Intestinal Health, Immune Modulation, and Ammonia-Nitrogen Stress Resilience in Hybrid Grouper

**DOI:** 10.3390/antiox13030317

**Published:** 2024-03-06

**Authors:** Congjie Han, Huizhong Shi, Congcong Cui, Jiawen Wang, Ling Li, Weilie Bei, Yan Cai, Shifeng Wang

**Affiliations:** 1 Collaborative Innovation Center of Marine Science and Technology, Hainan University, Haikou 570228, China; congjie_h113@163.com (C.H.); 15666933219@163.com (H.S.); 13569183135@163.com (C.C.); wangjiawen9599@163.com (J.W.); 18389716550@163.com (L.L.); 21220951340001@hainanu.edu.cn (W.B.); 2 Hainan Provincial Key Laboratory for Tropical Hydrobiology and Biotechnology, School of Marine Biology and Fisheries, Hainan University, Haikou 570228, China; 3 School of Life and Health Sciences, Hainan University, Haikou 570228, China

**Keywords:** *Bacillus subtilis*, hybrid grouper, growth, immunity, intestinal health, ammonia-nitrogen stress

## Abstract

In the dynamic field of intensive aquaculture, the strategic application of probiotics has become increasingly crucial, particularly for enhancing resistance to environmental stressors such as ammonia-nitrogen. Over a 42-day period, this study investigated the effects of different probiotic strains—*Bacillus subtilis* (BS, 6-3-1, and HAINUP40)—on the health and resilience of hybrid groupers. Each strain, distinct in its origin, was assessed for its influence on growth performance, antioxidant capacity, immune gene expressions, and ammonia-nitrogen stress response in the hybrid grouper. The experimental design included a control group and three experimental groups, each supplemented with 1 × 10^8^ CFU/g of the respective probiotic strains, respectively. Our results demonstrated notable differences in growth parameters, including final body weight (FBW) and feed conversion ratio (FCR). The 6-3-1 strain, originating from grouper, exhibited significant improvements in growth, oxidative capacity, and intestinal health. Conversely, the BS strain achieved the highest survival rates under ammonia-nitrogen stress, indicating its superior ability to regulate inflammatory responses despite its less pronounced growth-promoting effects. The HAINUP40 strain was distinguished for its growth enhancement and improvements in intestinal health, though it also showed significant activation of inflammatory genes and decreased resistance to ammonia-nitrogen stress after extended feeding. The uniqueness of this study lies in its detailed examination of the strain-specific effects of probiotics on fish in the context of ammonia-nitrogen stress, a significant challenge in contemporary aquaculture. The research revealed that host-derived probiotics, particularly the 6-3-1 strain, provided more comprehensive benefits for growth performance and stress resilience. In contrast, the BS and HAINUP40 strains exhibited varying efficiencies, with BS excelling in stress resistance and HAINUP40 promoting growth and gut health. In conclusion, this study underscores the complex roles of different probiotic strains in aquaculture, contributing to the understanding of probiotic applications and presenting new approaches to address the challenges of intensive farming.

## 1. Introduction

Aquaculture, a rapidly expanding sector within the global food industry, faces significant challenges regarding the health and sustainability of aquatic species [1]. Intensive farming methods have led to environmental degradation, notably through changes in water quality, such as increased levels of ammonia-nitrogen, total organic matter, and nitrites [2]. Excessive ammonia accumulation in aquatic environments directly impairs the gill function of aquatic animals, induces oxidative stress and metabolic disorders in fish, diminishes growth performance (such as feed conversion ratio and muscle quality), and increases mortality rates among cultured species [3,4,5]. Furthermore, runoff from nutrient-enriched aquaculture water contributes to the eutrophication of aquatic ecosystems by causing algal blooms [6,7,8,9]. These adverse factors pose a challenge to the continuously developing aquaculture industry, the environmental stress on aquatic environments, and the increasing demand for high-quality seafood products.

Traditionally, aquaculture has relied heavily on antibiotics and chemical agents to combat stress, diseases and promote growth [10,11]. However, concerns have arisen over the impacts of such practices on gut microbiota, the emergence of antibiotic-resistant strains, environmental pollution, and potential adverse effects on human health [12,13]. These issues necessitate a shift towards more natural and sustainable alternatives. In response to these challenges and the increasing demand for high-quality marine products, it is imperative to develop sustainable health management strategies for aquaculture [14]. These strategies should not only focus on growth performance but also provide effective solutions for environmental stressors, such as ammonia-nitrogen stress [15], thereby making significant contributions to global food security. With projections indicating a more intensive form of aquaculture by 2050, widespread concerns have arisen regarding the immune capabilities of aquatic animals and their resilience to environmental stressors [16,17].

In recent years, there has been a significant increase in the exploration of aquatic probiotics, particularly *Bacillus* species, due to their numerous benefits. Research has demonstrated that probiotics significantly enhance the activity of digestive enzymes in aquatic animals, such as proteases, amylases, and lipases [18]. Additionally, they improve gut health by modulating the intestinal microbiota [19] and bolstering immune function, thereby augmenting resistance to pathogens [20]. These synergistic effects not only improve feed conversion rates but also reduce feed input, thereby generating substantial economic benefits for the aquaculture industry [21,22]. Further evidence supports the utilization of *Bacillus* strains, including *Bacillus* strains such as *B. subtilis*, *B. velezensis*, and *B. tequilensis,* in hybrid groupers (*E. fuscoguttatus* ♀ × *E. lanceolatus* ♂) and Chinese perch (*Siniperca chuatsi*), corroborating the aforementioned benefits [23,24]. Additionally, probiotics can positively impact aquaculture water quality by consuming excess nutrients (such as phosphorus, nitrogen, and ammonia) in the rearing water, offering potential positive effects in suppressing harmful algal blooms and optimizing the aquaculture environment [25,26,27]. This offers evidence of the potential economic and environmental advantages of specific probiotics to improve water quality in intensive aquaculture applications.

Despite the widely recognized advantages of *Bacillus* probiotics in enhancing fish health, growth, improving water quality, and stress resilience, the strain-specific and time-dependent responses to ammonia stress in groups require further investigation. The antioxidant capacity and regulation of inflammatory genes are considered to play a pivotal role in counteracting oxidative stress induced by environmental factors, such as ammonia exposure and pathogen infections [28]. Hybrid groupers exposed to ammonia-nitrogen stress exhibit a significant reduction in the body’s antioxidant capacity and an increase in the expression of pro-inflammatory genes [28]. Research indicated that supplementation with strains like *B. subtilis*, *B. licheniformis*, *B. cereus*, and *P. marcusii* significantly boosts antioxidant capabilities, diminishes the expression of pro-inflammatory genes, and offers protection to grass carp and tilapia against oxidative stress from *Aeromonas hydrophila* infections [29,30,31]. These findings underscore the potential of these probiotic strains to enhance fish resilience to oxidative stress, thereby improving their overall health and survival.

In our previous research, we identified three potential *B. subtilis* probiotics (BS, 6-3-1, and HAINUP40) from different sources, finding that each exhibited distinct probiotic activity. The BS strain isolated from shrimp paste demonstrated high protease and amylase activities in vitro, suggesting potential benefits for nutrient digestion. The 6-3-1 strain from the hybrid grouper gut effectively promoted growth, enhanced non-specific immune responses, and increased disease resistance, showing pathogen inhibitory properties, indicating its role in enhancing gut health [32]. The HAINUP40 strain from natural pond water improved growth, digestive enzyme activity, non-specific immunity, and disease resistance in Nile tilapia and possessed self-aggregation and hydrophobicity traits, considered effective in nutrient absorption and immune modulation [33]. This 42-day study assessed these probiotics’ short-term and long-term impacts and their response to ammonia-nitrogen stress at 14, 28, and 42 days.

The hybrid grouper (*E. lanceolatus* ♂ × *E. fuscoguttatus* ♀) is a fast-growing, high-quality, economically valuable carnivorous marine fish, a primary species in China’s intensive aquaculture [34]. Thus, it serves as an excellent model for ammonia-nitrogen stress studies. This research investigated the impact of three *Bacillus* strains from diverse sources (shrimp paste, grouper gut, and pond water) on key growth parameters, serum antioxidant markers (GSH-Px, CAT, T-AOC, and MDA), intestinal structure, digestive enzyme activity, immune-related genes, and ammonia stress resistance in hybrid groupers. Uniquely, this study explored strains from natural and ecological sources and examined their effects on hybrid groups over different rearing periods (14, 28, 42 days), providing a comprehensive perspective on the potential benefits of probiotics in aquaculture. It evaluated whether host-derived strains are more effective than those from other sources. This study aims to offer valuable insights for aquaculture and probiotic research, focusing on improving grouper health and stress recovery, thus presenting practical solutions to challenges faced by the industry.

## 2. Materials and Methods

### 2.1. Experimental Setup and Acclimation

The experiment was conducted at a cooperative grouper aquaculture facility located in Haitou Town, Danzhou City, Hainan Province. A total of 360 healthy grouper fish were acclimated in four 1000-L tanks for 14 days prior to the feeding trial. During the acclimation period, fish were given ample time to adapt to their environment and were fed a balanced diet to meet their nutritional needs.

### 2.2. Preparation of Probiotic Diets

The three *Bacillus* strains (BS/6-3-1/HAINUP40) used in this experiment were isolated in our laboratory and have demonstrated probiotic effects. The BS strain exhibited high protease and amylase activity in vitro, suggesting potential benefits in nutrient digestion. The 6-3-1 strain showed pathogen inhibition properties, indicating its role in enhancing gut health [32]. The HAINUP40 strain, known for its self-aggregation and surface hydrophobicity, is considered effective in nutrient absorption and immune modulation [33]. Each *Bacillus* strain was cultured in LB liquid medium at 37 °C for 24 h, then centrifuged and resuspended in PBS. The suspensions were uniformly sprayed onto the surface of the base feed at final concentrations of 1 × 10^8^ CFU/g for BS, 6-3-1, and HAINUP40. The diets were labeled as Control (base diet + PBS), BS Diet (base diet + 1 × 10^8^ CFU/g *B. subtilis* BS), 6-3-1 Diet (base diet + 1 × 10^8^ CFU/g *B. subtilis* 6-3-1), and HAINUP40 Diet (base diet + 1 × 10^8^ CFU/g *B. subtilis* HAINUP40). After preparation, the diets were air-dried in a bacterial oven at 30 °C for 12 h. The control diet was sprayed only with PBS under the same proportions and conditions. Diets were prepared biweekly, dried, and then stored at −20 °C for future use. The basic diet’s ingredients and approximate composition are listed in Table 1.

### 2.3. Fish Rearing and Experimental Design

Prior to the start of the feeding trial, the grouper was fasted for 24 h. We measured their weight and length before randomly assigning 360 fish of similar weight (initial weight W_0_ = 532.28 ± 27.31 g) to 12 plastic tanks of 500 L each (*n* = 30 per tank). These were then divided into four groups (*n* = 3 replicates per group). A consistent light cycle was maintained in each tank, with equal intervals of 12 h of light and darkness. Additionally, each tank was equipped with an independent oxygenation system to ensure adequate oxygen levels.

The feeding trial was conducted over a period of 42 days, with sampling at 12, 28, and 42 days. Feeding occurred twice daily (at 09:00 and 17:00), with quantities sufficient to reach satiation. Uneaten feed and metabolic waste were collected and removed regularly after feeding, and the tanks were refreshed with aerated seawater every two days to ensure a comfortable external environment. Throughout the experiment, water quality parameters were maintained at a temperature of 26.50 ± 1.50 °C, dissolved oxygen at 6.50 ± 0.5 mg/L, pH at 7.6 ± 0.5, total ammonia-nitrogen (T-AN) at 0.26 ± 0.03 mg/L, and nitrites at 0.04 ± 0.01 mg/L.

### 2.4. Sampling Procedure

Sampling occurred on days 14, 28, and 42. Fish were fasted for 24 h prior to sampling and anesthetized with 80 mg/L tricaine methanesulfonate. Blood samples were obtained via caudal venipuncture and allowed to clot at room temperature for 30 min, followed by centrifugation at 4 °C and 3000 rpm for 10 min to separate the serum. Serum samples were stored at −80 °C until further analysis for antioxidant parameters. Liver samples were also collected post-dissection and stored at −80 °C for immune-related gene expression analysis. Mid-sections of the intestine were quickly and individually collected, divided into two parts: one flash-frozen in liquid nitrogen and stored at −80 °C for digestive enzyme activity analysis, and the other preserved in Bouin’s solution for 24 h for subsequent intestinal morphology analysis.

### 2.5. Growth Indices

Over the 42-day trial period, daily feed intake was measured. On days 14, 28, and 42, after a 12 h fasting period, the weight and length of each group were recorded. Growth indicators such as Weight Gain (WG), Average Daily Gain (ADG), Feed Conversion Ratio (FCR), and Condition Factor (CF) were calculated using the following formulas:

WG (g) = FBW_t_ − IBW
FCR (%) = WG (g)/Dry feed intake (g)
ADG (g) = WG (g)/days (d) × 100
CF (g/cm^3^) = FBW_t_ (g)/(FL)^3^ (cm)^3^ × 100
where IBW, FBW_t_, and FL refer to the initial weight, final weight, and final length, respectively.

### 2.6. Serum Antioxidant Activities and Malondialdehyde Levels

The serum antioxidant enzyme activities were determined using assay kits for Glutathione Peroxidase (GSH-PX), (colorimetric method, codes: A005-1-2), Catalase (CAT), (Visible light, codes: A007-1-1), Total Antioxidant Capacity (T-AOC), (ABTS method, codes: A015-2-1), and Malondialdehyde (MDA) (TBA method, codes: A003-1-2). The kits were procured from the Nanjing Jiancheng Bioengineering Institute, Nanjing, China.

### 2.7. Intestinal Morphology Parameters

Mid-intestinal samples fixed in Bouin’s solution were dehydrated in ethanol, cleared in xylene, infiltrated, and embedded in paraffin to form solid wax blocks. These embedded intestinal samples were then sectioned into approximately 4 μm thick slices using an automatic rotary microtome (Histo Core, Leica, Nussloch, Germany). Six fish per treatment group were selected. From each fish, a single cross-sectional area of the mid-intestine was prepared for pathological examination. The sections were stained with Hematoxylin and Eosin (H&E) and observed under an optical microscope (Nikon, Eclipse Ci-L, Tokyo, Japan). Analysis was conducted on representative cross-sectional areas of the mid-intestine of each fish. To obtain random histological areas, the intestinal section was divided into quadrants, with one random position within each quadrant selected for measurement. Photographs of each section were captured, and analysis software (Halo V.3.0.311.314, Indica Lab, Albuquerque, NM, USA) was used to measure villus length (VL), plica width (PW), and muscle thickness (MT). Goblet cell (GC) counts were performed in representative areas for goblet cell counting, with three measurements of goblet cell density taken across the representative intestinal villi in each section.

### 2.8. Intestinal Digestive Enzyme Activity

The activity of digestive enzymes was determined using the method described by [35]. Intestinal samples were thawed at 4 °C, weighed, and homogenized in a tissue homogenization medium (ratio: tissue (g): medium (v) = 1:9, pH = 7.6) using an automatic sample grinder (JXFSTPRP-L, Jingxin^®^, Shanghai, China) at 4 °C and 60 Hz/s for 2 min. After centrifugation at 4 °C and 2500 rpm for 10 min, the supernatant was collected and stored at −80 °C. The activities of gastric protease, trypsin, and lipase were further analyzed using assay kits from Nanjing Jiancheng Bioengineering Institute, Nanjing, China.

### 2.9. Hepatic Immune-Related Gene mRNA Expression

The expression levels of hepatic inflammation-related genes (TNFα, IL-1, IL-6, IL-10, TGFβ1, TLR3, TLR22, Myd88, IκBα, P65), and apoptosis genes (Bax, Bcl-2, Casp3, Casp8, Casp9) were measured. Approximately 100 mg of liver tissue was homogenized in 1 mL of Trizol, and RNA was extracted following the manufacturer’s instructions. RNA purity and concentration were determined at 260/280 nm using a NanoDrop1000 (Thermo Scientific, Waltham, MA, USA), and RNA integrity was confirmed by 1% agarose gel electrophoresis. cDNA was synthesized according to the Prime-ScriptTM manual and stored at −20 °C. Q-PCR was performed using SYBR Premix Ex TaqTM (TaKaRa Biotechnology, Dalian, China). Specific primers are listed in Table 2. The PCR protocol consisted of an initial step at 95 °C for 30 s, followed by 40 cycles of 95 °C for 5 s and 60 °C for 31 s. Relative gene expression was calculated using the 2^−ΔΔCt^ method with β-actin as the internal control.

### 2.10. Ammonia-Nitrogen Stress Test

Following a 42-day feeding trial, 45 fish of similar initial size from each experimental group were randomly selected for an ammonia-nitrogen stress test. During the 7-day stress test period, feeding was halted. Similar to the ammonia-nitrogen stress concentration determined by Cao et al. [28], for hybrid groupers, the preliminary experiment in our study established a 96-h LC50 under ammonia stress at 66.32 mg/L for hybrid groupers. We chose an ammonia concentration of 50% LC50 for 96 h for the experiment. A prepared stock solution of NH_4_Cl (10 g/L) was added to each 500 L tank to adjust the ammonia concentration to a stress level of 66.00 ± 0.86 mg/L. During the stress test, water temperature, pH, and ammonia concentration were measured every six hours. Mortality was recorded every six hours, and deceased fish were removed. The NH_4_Cl stock solution was used to maintain the required concentration by adjusting the non-ionic ammonia levels. The ammonia-nitrogen stress was monitored for seven days, with one-third of the test water being replaced every 12 h. Water parameters were maintained at a salinity of 30 ppt, temperature of 28 ± 2 °C, pH between 7.6 and 8.0, and dissolved oxygen levels at or above 6.6 mg/L.

### 2.11. Data Analysis

The experimental data were systematically recorded and organized using Microsoft Excel 2010. The data were initially analyzed with the Shapiro–Wilk Test to assess normality. For data conforming to a normal distribution, a one-way Analysis of Variance (ANOVA) was utilized. Statistical analysis was conducted using IBM SPSS software, version 21.0. For multiple comparisons, Tukey’s test was applied. The results were expressed as the mean ± SEM. For the challenge test, mortality data were analyzed using the Kaplan–Meier method. Differences between the control and probiotic-supplemented groups were assessed using the Mantel–Cox log-rank test. Statistical significance was determined based on a *p*-value threshold: a *p*-value greater than 0.05 was considered to indicate no significant difference, while a *p*-value less than 0.05 was indicative of statistical significance. In the results, columns marked with different letters (^a^, ^b^, ^c^, ^d^) signified statistically significant differences among the groups.

## 3. Results

### 3.1. Growth Performance

As presented in Table 3, the analysis of growth performance revealed significant differences among treatments over the 42-day feeding trial. There was no significant variation in initial body weight (IBW) across all groups. However, the final body weight (FBW) of groups treated with strains 6-3-1 and HAINUP40 showed a significant increase compared to the CON and BS groups, suggesting the potential growth-promoting effects of these specific probiotics (*p* < 0.001). This was reflected in WG, where the 6-3-1 and HAINUP40 groups significantly outperformed the CON and BS groups (*p* < 0.001). The improvement in FCR further corroborated this finding, with the HAINUP40 group demonstrating the most efficient feed utilization at all sampling points (14, 28, and 42 days), followed closely by the 6-3-1 strain. ADG was notably higher in the HAINUP40 group, indicating consistent growth benefits when this probiotic was administered (*p* < 0.05). However, CF did not exhibit a consistent pattern across treatments, suggesting that the influence of probiotics might be more pronounced on growth than on body composition.

### 3.2. Serum Antioxidant Activities and Malondialdehyde Levels

**Figure 1:**
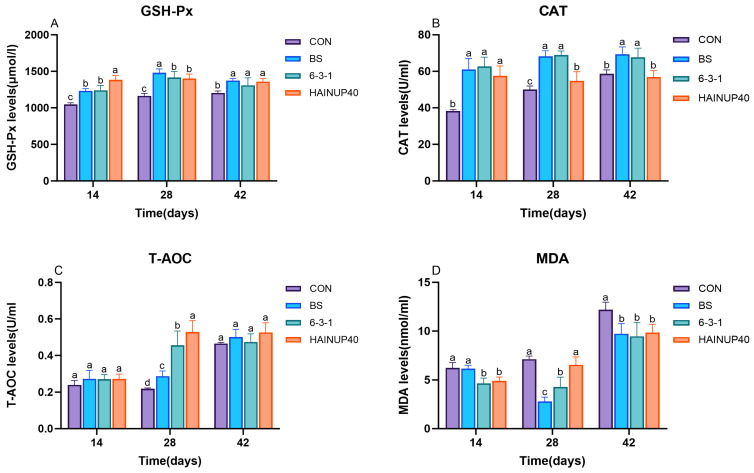
Effects of three *B. subtilis* strains on the serum antioxidant activities and malondialdehyde levels in the hybrid grouper. (**A**): glutathione peroxidase (GSH-Px); (**B**): catalase (CAT); (**C**): total antioxidant capacity (T-AOC); (**D**): malondialdehyde (MDA). The values are presented as the mean ± SEM (*n* = 9). Columns marked with different letters (^a^, ^b^, ^c^, ^d^) indicate statistically significant differences between groups, as determined by ANOVA and Tukey’s test (*p* < 0.05).

As illustrated in Figure 1, the analysis of serum antioxidant indices indicated that the antioxidant capacity in the hybrid grouper changed over different feeding periods. On day 14, compared to the CON group, the BS, 6-3-1, and HAINUP40 groups showed a significant increase in serum GSH-Px and CAT levels (*p* < 0.05). Notably, HAINUP40 demonstrated a more pronounced effect on GSH-Px compared to the BS and 6-3-1 groups. Both 6-3-1 and HAINUP40 significantly reduced MDA levels (*p* < 0.05). On day 28, compared to CON, the BS, 6-3-1, and HAINUP40 groups significantly enhanced serum GSH-Px, CAT, and T-AOC levels (*p* < 0.05). The BS group exhibited the highest improvement in GSH-Px. In terms of enhancing CAT levels, BS and 6-3-1 outperformed HAINUP40, with both groups significantly reducing serum MDA levels (*p* < 0.05), and BS showing better efficacy than 6-3-1. Relative to BS and 6-3-1, HAINUP40 most effectively elevated serum T-AOC levels, with 6-3-1 being superior to BS and both significantly higher than the CON group (*p* < 0.05). On day 42, compared to CON, the BS, 6-3-1, and HAINUP40 groups significantly increased serum GSH-Px levels and markedly reduced serum MDA levels (*p* < 0.05). The BS and 6-3-1 groups significantly enhanced serum CAT levels (*p* < 0.05).

### 3.3. Intestinal Morphology

**Figure 2:**
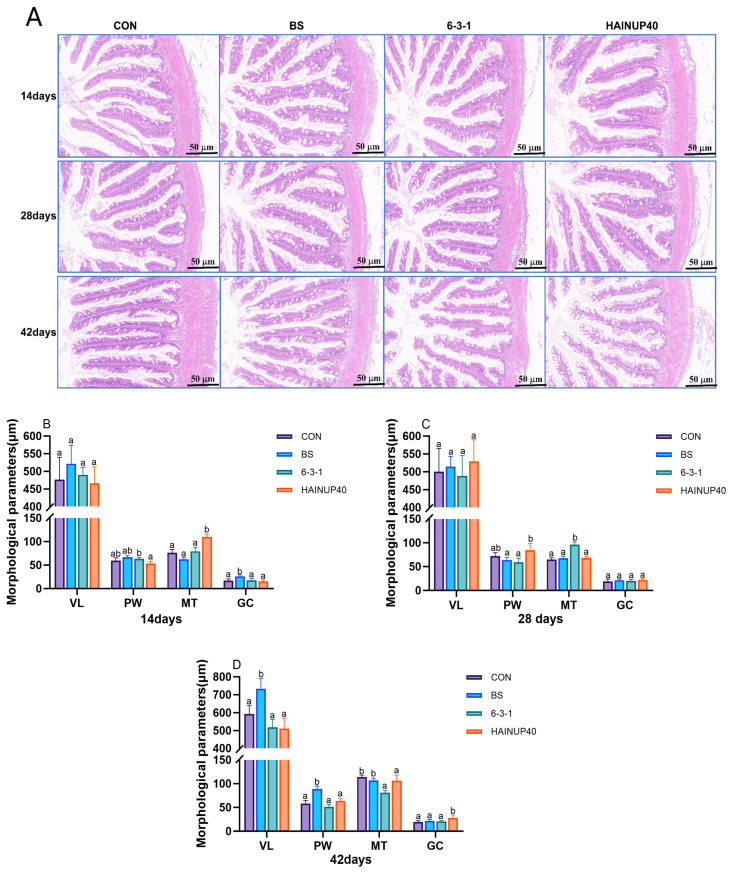
Effects of three *B. subtilis* strains on the intestinal morphology. (**A**): intestinal sections (H&E Staining) at 14, 28, and 42 days; (**B**): intestinal morphology at 14 days; (**C**): intestinal morphology at 28 days; (**D**): intestinal morphology at 42 days. The thickness of the analyzed sections was maintained at 4 μm, with cross-sectional diameters measured at 14 days (1426 ± 84 μm), 28 days (1607.48 ± 200.97 μm), and 42 days (1661.88 ± 53.85 μm). The scale bar is 50 μm. *VL*: villus length; *PW*: plica width; *MT*: muscle thickness; *GC*: goblet cell. The values are presented as the mean ± SEM (*n* = 6). Columns marked with different letters (^a^, ^b^) indicate statistically significant differences between groups, as determined by ANOVA and Tukey’s test (*p* < 0.05).

As shown in Figure 2, the analysis of intestinal morphology revealed changes in the gut structure of hybrid groupers over different feeding periods. On day 14, compared to CON, HAINUP40 significantly increased MT, and BS notably increased GC count (*p* < 0.05). Compared to HAINUP40, 6-3-1 significantly enhanced PW (*p* < 0.05). On day 28, compared to CON, the 6-3-1 group significantly increased MT (*p* < 0.05). Relative to BS and 6-3-1, the HAINUP40 group significantly elevated PW (*p* < 0.05). On day 42, compared to CON, BS significantly increased villus length in VL and PW (*p* < 0.05). The 6-3-1 and HAINUP40 groups significantly reduced MT (*p* < 0.05). HAINUP40 notably increased the GC count in the intestine (*p* < 0.05).

### 3.4. Intestinal Digestive Enzyme Activity

**Figure 3:**
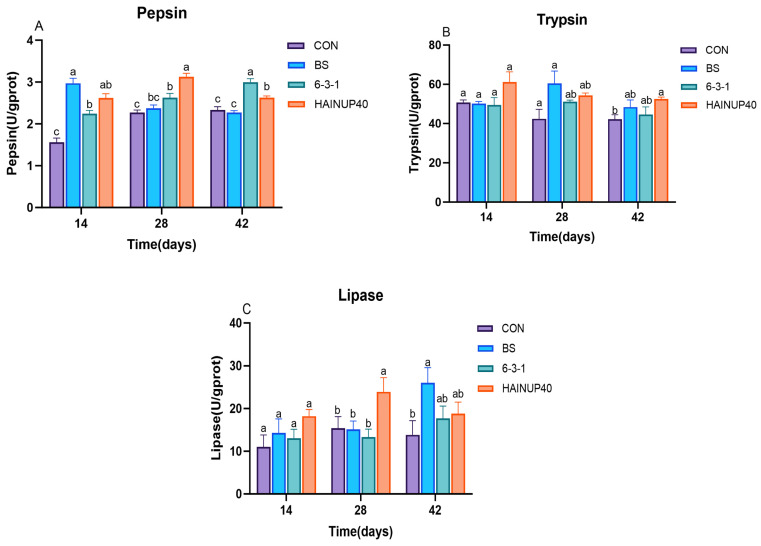
Effects of three *B. subtilis* strains on the digestive enzyme activities. (**A**): pepsin; (**B**): trypsin; (**C**): lipase. U: one unit of enzyme activity; mg prot: mg protein; g prot: g protein. The values are presented as the mean ± SEM (*n* = 9). Columns marked with different letters (^a^, ^b^, ^c^) indicate statistically significant differences between groups, as determined by ANOVA and Tukey’s test (*p* < 0.05).

As depicted in Figure 3, the analysis of intestinal digestive enzyme activity showed changes in the hybrid group over various feeding periods. On day 14, compared to the CON group, the BS, 6-3-1, and HAINUP40 groups significantly increased levels of intestinal pepsin, with the BS group showing the most pronounced effect (*p* < 0.05). By day 28, compared to CON, the 6-3-1 and HAINUP40 groups significantly enhanced intestinal pepsin levels, with HAINUP40 outperforming 6-3-1 (*p* < 0.05). Additionally, HAINUP40 significantly elevated lipase levels (*p* < 0.05). On day 42, compared to CON, the 6-3-1 and HAINUP40 groups significantly increased intestinal pepsin levels, with HAINUP40 demonstrating superior efficacy (*p* < 0.05). Moreover, HAINUP40 significantly raised trypsin levels, while BS also enhanced lipase levels. (*p* < 0.05).

### 3.5. Expression of Immune-Related Genes in the Liver

**Figure 4:**
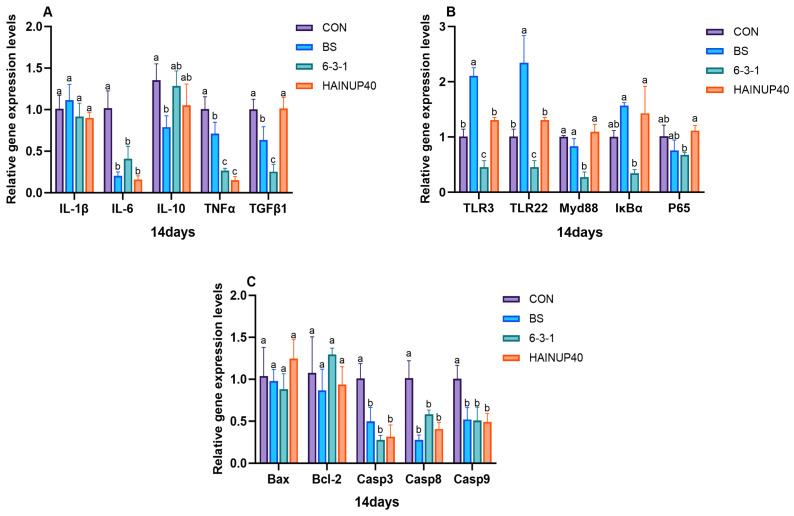
Effects of three *B. subtilis* strains on the expression of immunity-related genes in the hybrid grouper liver (14 days). (**A**): genes related to inflammatory factors; (**B**): genes associated with inflammation pathways; (**C**): genes related to apoptotic proteins. The values are presented as the mean ± SEM (*n* = 9). Columns marked with different letters (^a^, ^b^, ^c^) indicate statistically significant differences between groups, as determined by ANOVA and Tukey’s test (*p* < 0.05).

**Figure 5:**
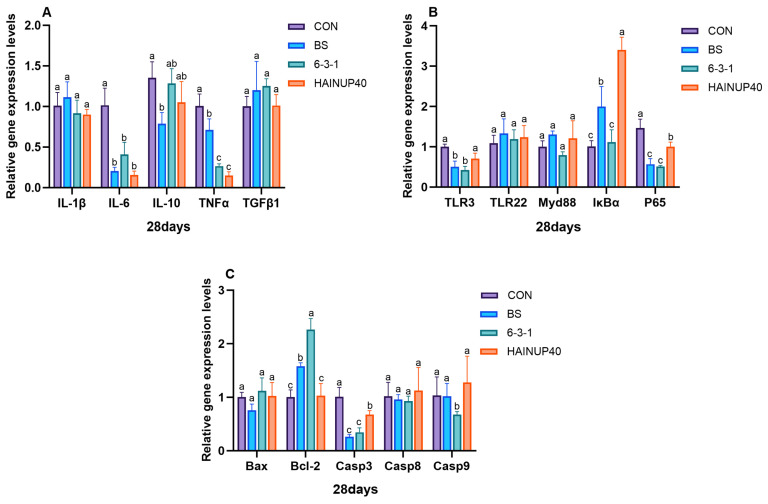
Effects of three *B. subtilis* strains on the expression of immunity-related genes in hybrid grouper liver (28 days). (**A)**: genes related to inflammatory factors; (**B**): genes associated with inflammation pathways; (**C**): genes related to apoptotic proteins. The values are presented as the mean ± SEM (*n* = 9). Columns marked with different letters (^a^, ^b^, ^c^) indicate statistically significant differences between groups, as determined by ANOVA and Tukey’s test (*p* < 0.05).

**Figure 6:**
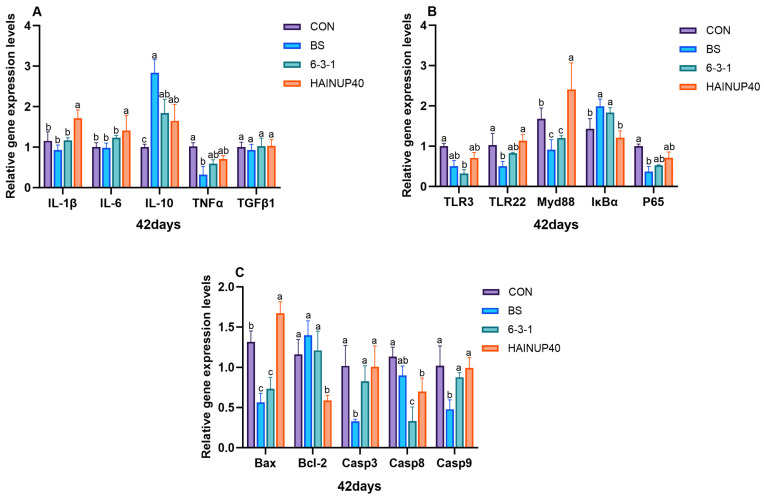
Effects of three *B. subtilis* strains on the expression of immunity-related genes in hybrid grouper liver (42 days). (**A**): genes related to inflammatory factors; (**B**): genes associated with inflammation pathways; (**C**): genes related to apoptotic proteins. The values are presented as the mean ± SEM (*n* = 9). Columns marked with different letters (^a^, ^b^, ^c^) indicate statistically significant differences between groups, as determined by ANOVA and Tukey’s test (*p <* 0.05). IL-1β: interleukin-1 beta; IL-6: interleukin-6; IL-10: interleukin-10; TNFα: tumor necrosis factor alpha; TGFβ1: transforming growth factor beta-1; TLR3: toll-like receptor 3; TLR22: toll-like receptor 22; Myd88: myeloid differentiation primary response 88; IκBα: nuclear factor of kappa light polypeptide gene enhancer in B-cells inhibitor, alpha; P65: nuclear factor kappa b subunit p65; Bax: Bcl2 associated x, apoptosis regulator; Bcl-2: B-cell lymphoma 2; Casp3: apoptosis-related cysteine peptidase; Casp8: apoptosis-related cysteine peptidase; Casp9: apoptosis-related cysteine peptidase.

As shown in Figure 4, on day 14, the analysis of liver immune-related genes revealed that, compared to CON, BS reduced the expression levels of IL-10 and TGFβ1 genes (*p* < 0.05). Also, 6-3-1 lowered TGFβ1, TLR3, TLR22, and Myd88 gene expression levels (*p* < 0.05). Additionally, the BS, 6-3-1, and HAINUP40 groups significantly decreased IL-6, TNFα, Casp3, Casp8, and Casp9 gene expression levels (*p* < 0.05). As presented in Figure 5, on day 28, compared to CON, BS significantly reduced IL-10, TLR-3, and P65 expression levels and increased IκBα and Bcl-2 gene expression (*p* < 0.05). Moreover, 6-3-1 markedly lowered TLR3, P65, and Casp9 expression levels and increased Bcl-2 gene expression (*p* < 0.05). HAINUP40 significantly elevated IκBα expression (*p* < 0.05). Furthermore, BS, 6-3-1, and HAINUP40 groups all significantly decreased IL-6, TNFα, and Casp3 expression levels (*p* < 0.05). As indicated in Figure 6, on day 42, compared to CON, BS significantly lowered TNFα, TLR-22, P65, Bax, Casp3, and Casp9 expression levels and raised IκBα and Bcl-2 gene expression (*p* < 0.05). Additionally, 6-3-1 significantly decreased TLR3, Bax, and Casp8 expression levels and increased Myd88 gene expression (*p* < 0.05). HAINUP40 notably raised IL-6, Myd88, IκBα, and Bax expression levels and decreased Bcl-2 and Casp8 expression (*p* < 0.05). Additionally, the BS, 6-3-1, and HAINUP40 groups all significantly increased IL-10 expression levels (*p* < 0.05).

### 3.6. Ammonia-Nitrogen Stress Test

**Figure 7:**
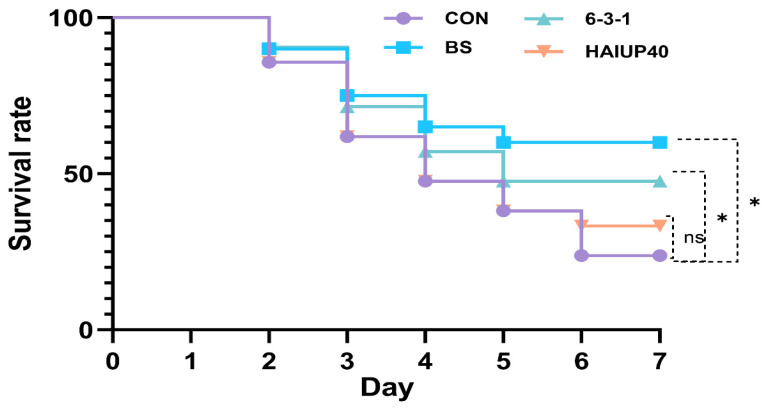
The Kaplan–Meier survival analysis of hybrid grouper-fed control and probiotic-supplemented groups after an ammonia-nitrogen stress test. Differences between the control and probiotic-supplemented groups were assessed using the Mantel–Cox log-rank test. “*” represents significant differences. “ns” indicates no significant difference. * *p* < 0.05.

The results of the ammonia-nitrogen stress test, as depicted in Figure 7, showed that the survival rates of the BS and 6-3-1 groups were significantly higher than those of the control group. Specifically, the survival rate was 60.0% for the BS group and 47.619% for the 6-3-1 group (*p* < 0.05). In contrast, the survival rate of the HAINUP40 group (33.33%) did not differ significantly from the control group (23.81%, *p >* 0.05). These results suggest that the BS and 6-3-1 groups enhanced the resistance of the hybrid grouper to ammonia-nitrogen stress, increasing survival rates under stress, while HAINUP40 did not demonstrate a significant effect.

## 4. Discussion

In aquaculture, the application of probiotics has become a key strategy to enhance fish growth performance, bolster immunity, and increase resistance to pathogens [36]. Despite widespread recognition and research into these benefits, in-depth studies on their physiological impacts on aquatic animals at different growth stages (such as 14, 28, and 42 days) remain relatively scarce. Particularly under environmental stresses like ammonia-nitrogen, understanding the mechanisms of action and antioxidant capabilities of probiotics is crucial. With the rapid development of intensive aquaculture, ammonia-nitrogen stress has become a significant factor affecting farming efficiency and fish survival [37]. Therefore, exploring the potential role of probiotics in alleviating such stress not only addresses current challenges in aquaculture but also opens new research avenues for their future application.

Supplementing diets with probiotics has been shown to improve growth performance in various fish species, including the Caspian kutum, olive flounder, tilapia, and Pacific red snapper [38,39,40,41]. In our study, we evaluated the impact of *B. subtilis* strains from different sources (shrimp paste, grouper gut, and pond water) on the growth performance of hybrid groupers (*E. lanceolatus* ♂ × *E. fuscoguttatus* ♀). Over a 42-day trial period, groups supplemented with strains 6-3-1 and HAINUP40 exhibited enhanced growth performance compared to the CON and BS groups. These groups showed significantly higher FBW and WG, indicating the potent growth-promoting effects of these specific probiotic strains (*p* < 0.001). Furthermore, FCR data indicated higher feed utilization efficiency in the HAINUP40 group at all sampling points (14, 28, and 42 days), followed by the 6-3-1 strain. Notably, ADG was significantly higher in the HAINUP40 group, suggesting consistent growth advantages when using this probiotic (*p* < 0.05). However, the CF values across different treatments did not exhibit a consistent pattern, implying that the impact of probiotics on growth may not be synchronous with changes in body condition. Similar studies have shown that the improvement in growth performance by probiotics can vary across different feeding periods. For instance, in tilapia diets, the addition of *B. pumilus* for 28–56 days significantly altered weight gain and final weight compared to earlier parameters. After 42 days of feeding, the inclusion of *Lactobacillus plantarum* E2 significantly increased the final weight, length, and weight gain in the yellow croaker [42]. In the diet of grouper *E. coioides*, probiotic supplementation also enhanced weight gain and feed efficiency [43]. However, some studies indicated that certain probiotic strains do not positively impact the growth performance of Nile tilapia. After a 21-day growth trial, tilapia-fed diets supplemented with *B. subtilis* showed no significant changes in growth performance [44]. Interestingly, experiments have also revealed that the addition of *B. amyloliquefaciens* to tilapia diets showed no significant growth-promoting effects after 30 days of feeding, but the benefits became apparent after 60 days. These findings highlighted the potential of specific probiotic strains to improve growth performance, with their regulatory effects on growth potentially varying across different feeding periods.

Serum antioxidant indicators such as GSH-Px, CAT, and T-AOC are crucial parameters for assessing the efficacy of an organism’s antioxidant defense system [45]. These indicators reflect the organism’s response to oxidative stress and are key factors in maintaining physiological balance and preventing disease [46]. Experimental results indicated significant variations in serum antioxidant indicators in the hybrid group over different periods of probiotic feeding. Compared to the control group, groups fed with BS, 6-3-1, and HAINUP40 showed significant increases in GSH-Px and CAT levels on day 14, with the HAINUP40 group exhibiting the most notable enhancement in GSH-Px. Additionally, the 6-3-1 and HAINUP40 groups significantly reduced MDA levels, indicating the effectiveness of probiotics in reducing oxidative stress. By day 28, all probiotic-supplemented groups had significantly elevated levels of GSH-Px, CAT, and T-AOC. The BS group displayed superior performance in enhancing GSH-Px, while BS and 6-3-1 were more effective in increasing CAT levels, and both significantly lowered MDA levels. Notably, the HAINUP40 group showed the greatest improvement in serum T-AOC levels. By day 42, compared to CON, all probiotic groups significantly enhanced GSH-Px levels and reduced MDA levels, with BS and 6-3-1 showing significant improvements in CAT levels. Similar short-term studies align with our findings. In gilthead seabream diets supplemented with *B.subtilis*, an increase in GSH-Px levels was observed [47]. After 60 days of feeding *Labeo rohita* a diet containing *L. plantarum* VSG3, significant increases in serum SOD and GSH-Px levels were noted [48]. In the diet of *Oreochromis mossambicus* supplemented with *B. licheniformis*, significant rises in serum GSH-Px and SOD were recorded at both 14 and 28 days [31]. These results suggest that probiotics enhance the overall health of aquatic animals by boosting their antioxidant capabilities. Furthermore, our study interestingly shows that probiotics have a significant and time-dependent positive impact on the antioxidant indicators in hybrid groups.

Intestinal structures, including MT, VL, GC count, and PW, play a crucial role in nutrient absorption and overall health in fish [49]. These parameters are indicative of intestinal health and functionality, which are essential for optimal growth and disease resistance in aquaculture species [50]. Our study demonstrated significant changes in the intestinal morphology of hybrid groupers under various probiotic treatments. On day 14, compared to the control group, the HAINUP40 group showed a significant increase in MT, while the BS group exhibited a notable increase in GC count. The 6-3-1 group displayed a significant enhancement in PW when compared to HAINUP40. By day 28, compared to CON, the 6-3-1 group significantly increased MT, and relative to BS and 6-3-1, HAINUP40 showed a marked improvement in PW. On day 42, compared to CON, BS significantly increased VL and PW, while 6-3-1 and HAINUP40 significantly reduced MT. HAINUP40 notably increased the GC count in the intestine. These results suggested that specific probiotics selectively influence various aspects of intestinal morphology in hybrid groups over different feeding periods. Similar effects have been observed in other studies. For instance, adding *L. rhamnosus* to tilapia diets for 30 days significantly promoted intestinal health, increasing the height and width of intestinal folds [41]. In carp diets supplemented with *B. coagulans* for 42 days, significant increases in MT and VL were noted, maintaining intestinal health. In *Channa argus* diets supplemented with *Lactococcus lactis*, an increase in villi density and more distinct and complete morphological structures were observed, along with increased MT and plica height and width [35]. These findings demonstrate that probiotic supplementation can improve intestinal structure. Furthermore, studies show that butyrate produced by bacteria can alleviate soy glycine-induced intestinal inflammation in hybrid groupers and promote villus proliferation and crypt deepening [51]. This research indicates that the metabolic products produced by specific probiotics can effectively alter intestinal structures, suggesting that the improvements in gut health are due to the strain-specific metabolic outputs of the probiotics.

Digestive enzymes, such as pepsin, trypsin, and lipase, are crucial for the effective digestion and assimilation of nutrients in fish [52]. These enzymes facilitate the breakdown of proteins, lipids, and carbohydrates, which are essential for fish growth and health [53]. Therefore, assessing the levels of these enzymes can provide insights into the digestive health of fish. Our results indicate significant changes in the levels of digestive enzymes in the hybrid grouper during different periods of probiotic feeding. By day 14, compared to the control group (CON), all probiotic groups (BS, 6-3-1, HAINUP40) showed a significant increase in intestinal pepsin levels, with the BS group exhibiting the most pronounced effect. On day 28, the 6-3-1 and HAINUP40 groups showed a significant increase in intestinal pepsin levels, with HAINUP40 outperforming 6-3-1. Additionally, HAINUP40 significantly elevated lipase levels. By day 42, the pepsin levels in the 6-3-1 and HAINUP40 groups continued to be significantly higher than CON, with HAINUP40 being more effective. Moreover, HAINUP40 significantly raised trypsin levels, and BS also increased lipase levels. Similar to our study, the addition of *Bacillus subtilis* to the diet of black tiger shrimp (*Penaeus monodon*) for 50 days significantly enhanced intestinal digestive enzyme (lipase) activity [54]. Likewise, in diets supplemented with *lactobacillus* for Channa argus fed for 56 days, there was a significant increase in intestinal protease, amylase, and lipase activities [35]. In grouper (*Epinephelus coioides*)-fed diets supplemented with *Lactobacillus* or *Enterococcus faecium*, protease activity was significantly enhanced [55]. These findings are consistent with our observations of increased pepsin and lipase levels in the probiotic treatment groups. This further corroborates the beneficial effects of probiotics on fish digestive health observed in our study. Additionally, the increased digestive enzyme activity in hybrid grouper-fed diets supplemented with the three probiotic groups across different feeding periods may explain the differential growth performance exhibited by the various feeding groups over time.

In this study, we focused on the regulatory effects of probiotics on liver immune gene expression. Specifically, we measured key immune genes including pro-inflammatory factors (IL-1β, IL-6, TNFα), anti-inflammatory factors (IL-10, TGFβ1), and genes related to inflammation and apoptosis (TLR3, TLR22, Myd88, IκBα, P65, Bcl-2, Bax, Casp3, Casp8, and Casp9). These genes play a critical role in regulating immune responses and maintaining health in fish liver [46,56], involving various immune pathways such as anti-inflammatory reactions and apoptosis, and are important indicators of fish immune health [57]. Our results revealed significant changes in the expression of liver immune genes in the hybrid group under different probiotic treatments. On day 14, compared to CON, the BS group showed a significant decrease in the expression of IL-10 and TGFβ1, while the 6-3-1 group reduced the expression of TGFβ1, TLR3, TLR22, and Myd88. All probiotic groups significantly lowered the expression of IL-6, TNFα, Casp3, Casp8, and Casp9, indicating early-stage immune gene regulation by probiotics. On day 28, the BS group further reduced the expression of IL-10, TLR-3, and P65 and increased IκBα, Bcl-2 expression; the 6-3-1 group lowered TLR3, P65, and Casp9 while elevating Bcl-2; HAINUP40 increased IκBα expression and decreased P65, Casp3, and Casp9. These results reveal the dynamic changes in immune gene regulation caused by probiotics. On day 42, the BS group reduced the expression of TNFα, TLR-22, Myd88, P65, Bax, Casp3, Casp9, and increased IκBα, Bcl-2; the 6-3-1 group lowered TLR3, Myd88, Bax, Casp8, while enhancing IκBα; HAINUP40 elevated IL-1β, IL-6, Myd88, Bax, and decreased IκBα, Bcl-2, Casp8. All groups enhanced IL-10 expression, demonstrating the long-term effects of probiotics on immune gene regulation. Similar studies support strain-specific probiotic impacts on immune responses at different feeding periods. For instance, *B. coagulans* BC1 in the carp diet showed significant changes in nonspecific immune genes IL-10, IL-1β, TNF-α, and TGF-β1mRNA expression levels [58]. *Pediococcus acidilactici* MA18/5M in trout diet significantly increased pro-inflammatory cytokines IL-1β and IL-8 while downregulating anti-inflammatory factor IL-10 [59]. *B. amyloliquefaciens* COFCAU_P1 in the *L. rohita* diet elevated pro-inflammatory genes IL-1β and TNFα in liver tissue [60]. These studies corroborate the strain-specific impacts of probiotics, which may be negative, as observed in our study, where HAINUP40 significantly elevated inflammatory gene expression on day 42. Furthermore, studies in hybrid groups (*E. fuscoguttatus* ♀ × *E. lanceolatus* ♂) with host-derived *B. subtilis* found significant upregulation of IL1β, IL6, MyD88, and IL10 genes, similar to our HAINUP40 immune regulation findings. These studies confirm the dynamic nature of probiotic regulation of immune gene expression, demonstrating varying potential across different feeding periods, especially in fish.

Numerous studies have demonstrated that the inclusion of probiotics such as *B. subtilis* [24], *B. amyloliquefaciens* [60], and *Lactobacillus* [43] in diets could significantly enhance the host’s resistance to pathogens. However, research on probiotics’ role in stress resistance remains relatively limited. Given the demand for high-quality protein in intensive aquaculture, ammonia-nitrogen stress has emerged as a significant environmental stressor, inducing oxidative stress and reducing resistance in farmed animals [61,62]. Some studies have indicated that probiotics regulate inflammation-related genes (such as IL-10, TNFα, TLR, etc.), thereby enhancing the host’s immune response and resistance to environmental stress [63,64]. This suggests that oxidative stress, triggered by environmental stressors, may activate downstream responses in inflammation pathways, potentially accelerating tissue damage and cell apoptosis [65,66]. According to our day 42 experimental results, the three probiotic groups (BS, 6-3-1, and HAINUP40) displayed varying protective effects against ammonia-nitrogen stress. The BS and 6-3-1 groups showed higher survival rates, likely due to their more effective enhancement of antioxidant enzyme activities and regulation of immune gene expressions. For instance, these groups more effectively increased CAT activity and downregulated expression of inflammatory pathways and apoptosis-related immune genes, thereby improving resistance to ammonia-nitrogen stress. In contrast, the HAINUP40 group’s effect was less significant than BS and 6-3-1, possibly due to weaker regulation of these antioxidant and immune-related indicators. Studies show that ammonia-nitrogen stress induces the maturation of pro-inflammatory cytokines in Nile tilapia, including TNF, IL1β, and IL6, activating oxidative stress and cellular damage leading to death [4]. New evidence also suggests that ammonia-nitrogen stress in hybrid groupers not only activates the expression of pro-inflammatory cytokines (TNF-α, IL-1β, IL-6, and IL-8) but also activates apoptotic proteins (Casp1, Casp8, and Casp9), promoting apoptosis [18]. These findings corroborate our experimental results, indicating that specific probiotic strains can enhance fish resistance to environmental stress (ammonia-nitrogen stress), primarily due to their regulatory effects on antioxidant enzymes and inflammatory genes.

## 5. Conclusions

This study explored the diverse beneficial effects of three distinct probiotic strains on growth enhancement and resistance to ammonia-nitrogen stress in intensive aquaculture. Our results revealed that different strains of the same bacterial species, originating from various sources, possess unique probiotic properties. Specifically, the host-derived 6-3-1 strain demonstrated the most comprehensive benefits in terms of growth performance and resistance to ammonia-nitrogen stress. During the 42-day experimental period, the BS strain showed superior resilience to ammonia-nitrogen stress, increasing the fish survival rate by 151.9% compared to the control group. Additionally, the HAINUP40 strain significantly enhanced growth performance, with a 13.3% increase in weight gain (WG) and a 35.11% reduction in feed conversion ratio (FCR), alongside improvements in intestinal health compared to the control group. These findings suggest strategic probiotic combinations could optimize growth and stress management in aquaculture, offering a novel approach to mitigating environmental impacts. While specific improvements and resistance percentages are subject to further quantification, this research paves the way for using host-derived probiotics to enhance aquaculture sustainability. However, we recognized the variability in the effectiveness of bacterial strains across different species and ecosystems, underscoring the necessity for more extensive application studies to fully harness the potential of probiotics in aquaculture.

## Figures and Tables

**Table 1:** Ingredients and approximate composition of a basic diet.

Ingredients	%
Red fishmeal	40.00
Casein	11.54
Gelatine	2.85
Wheat flour	20.00
Fish oil	4.66
Soy lecithin	2.00
Calcium monophosphate	1.00
Vitamin premix ^a^	0.22
Mineral premix ^b^	0.56
Antioxidants	0.06
Choline chloride	0.50
Vitamin C	0.09
Cellulose microcrystalline	16.52
Proximate composition (in dry matter)	
Crude protein	48.86
Crude lipid	10.75
Moisture	9.58

Vitamin premix composition (g/kg) ^a^: vitamin B_1_: 18.50 g; vitamin B_2_: 18.67 g; vitamin B_6_: 32.43 g; vitamin B_12_: 0.17 g; vitamin K: 3.43 g; vitamin E: 65.95 g; retinyl acetate: 6.77 g; VD: 34.33 g; nicotinic acid: 68.33 g; D-calcium pantothenate: 39.87 g; biotin: 18.53 g; folic acid: 3.97 g; inositol: 101.04 g; cellulose: 598.62 g; the rest ingredient was corn starch. Mineral premix composition (mg/g) ^b^: AlCl_3_·6H_2_O: 1 g; CoCl_2_·6H_2_O: 0.1 g; CaCO_3_: 350 g; CuCl_2_·2H_2_O: 1 g; FeSO_4_·7H_2_O: 2 g; KH_2_PO_4_: 200 g; MgSO_4_·7H_2_O: 10 g; MnSO_4_·7H_2_O: 2 g; NaH_2_PO_4_·H_2_O: 200 g; NaCl: 12 g; KF: 1 g; NaMoO_4_·2H_2_O: 0.5 g; NaSeO_3_: 0.4 g; KI: 0.1 g; zeolite powder: 219.9 g.

**Table 2:** Sequences of primers for qPCR.

Gene	Primer Name	Primer Sequence 5′–3′	Accession No.
IL-1β	IL-1β-F	CGACATGGTGCGGTTTC	XM_049578640.1
	IL-1β-R	TCTGTAGCGGCTGGTGG	
IL-6	IL-6-F	AGGAAGTCTGGCTGTCAGGA	XM_049603149.1
	IL-6-R	GCCCTGAGGCCTTCAAGATT	
IL-10	IL-10-F	ACACAGCGCTGCTAGACGAG	XM_049580695.1
	IL-10-R	GGGCAGCACCGTGTTCAGAT	
TNFα	TNFα-F	GTGGCCTACACGACTGCACC	XM_049582852.1
	TNFα-R	TACAAAGGGCCACAGTGAGA	
TGFβ1	TGFβ1-F	AACATCCCGCTACCTCGCTT	XM_049576571.1
	TGFβ1-R	TCCGCTCATCCTCATTCCCT	
TLR3	TLR3-F	TTCTTAACCATTCGCCCTCC	XM_033624878.1
	TLR3-R	GGCCCATATTGCTTCCATC	
TLR22	TLR22-F	TGTGACGGACAAACCGTGAT	JQ965995.1
	TLR22-R	GCGCATATGAGTCCCTTCCC	
IκBα	IκBα-F	GCCAGCAGCACATCACTTCC	XM_049590191.1
	IκBα-R	AGCCACCGTAGTTCAAGCAGTT	
Myd88	Myd88-F	CGAGCCAGGTAAACCCATCA	XM_049565206.1
	Myd88-R	CTCATCAAACAGGCGGAAGC	
P65	P65-F	GGGTGTGTATGGATGGGG	XM_049567828.1
	P65-R	TGGCTGGGTGGGTCTTAG	
Bax	Bax-F	CTCCCGAGCTACACTAGACA	XM_049590191.1
	Bax-R	GCATAGGGATCATGGGGGTG	
Bcl-2	Bcl-2-F	TTAGGTCGCAGTGAGT	XM_033637342.1
	Bcl-2-R	CATAGATGGGGAAGAG	
Casp3	Casp3-F	CGCAAAGAGTAGCGACGGA	XM_049571989.1
	Casp3-R	CGATGCTGGGGAAATTCAGAC	
Casp8	Casp8-F	TGCTTCTTGTGTCGTGATGTTG	XM_049598079.1
	Casp8-R	GCGTCGGTCTCTTCTGGTTG	
Casp9	Casp9-F	TTTTCCTGGTTATGTTTCGTGG	XM_033629367.1
	Casp9-R	TTGCTTGTAGAGCCCTTTTGC	
β-actin	β-actin-F	TACGAGCTGCCTGACGGACA	XM_049560987.1
	β-actin-R	GGCTGTGATCTCCTTCTGC	

**Table 3:** Effects of three *B. subtilis* strains on the growth parameters of hybrid groupers.

	Group				*p*-Value
Item	Control	BS	6-3-1	HAINUP40	
IBW, g	540.80 ± 23.17	531.67 ± 37.51	531.33 ± 20.55	525.33 ± 28.07	0.198
FBW, g	1344.00 ± 39.40 ^b^	1323.33 ± 24.34 ^b^	1422.00 ± 14.11 ^a^	1435.33 ± 21.55 ^a^	<0.001
**14 days**					
WG, g	331.87 ± 12.43 ^c^	313.67 ± 10.69 ^c^	346.67 ± 9.02 ^b^	422.67 ± 40.13 ^a^	<0.001
FCR, %	0.84 ± 0.04 ^a^	0.69 ± 0.02 ^c^	0.75 ± 0.05 ^b^	0.59 ± 0.03 ^d^	<0.001
ADG, g	22.12 ± 0.83 ^bc^	20.91 ± 0.71 ^c^	23.11 ± 0.60 ^b^	28.18 ± 2.68 ^a^	0.219
CF, g/cm^3^	0.67 ± 0.09 ^a^	0.70 ± 0.05 ^a^	0.65 ± 0.09 ^a^	0.47 ± 0.06 ^b^	0.103
**28 days**					
WG, g	510.20 ± 10.81 ^c^	489.33 ± 19.09 ^c^	558.33 ± 11.53 ^b^	616.00 ± 43.09 ^a^	<0.001
FCR, %	0.91 ± 0.01 ^a^	0.80 ± 0.02 ^b^	0.60 ± 0.02 ^c^	0.58 ± 0.02 ^c^	<0.001
ADG, g	20.41 ± 0.43 ^c^	19.57 ± 0.76 ^c^	22.33 ± 0.06 ^b^	24.64 ± 1.72 ^a^	0.023
CF, g/cm^3^	0.81 ± 0.07 ^a^	0.67 ± 0.08 ^c^	0.68 ± 0.04 ^c^	0.73 ± 0.05 ^bc^	<0.001
**42 days**					
WG, g	803.20 ± 38.30 ^b^	791.67 ± 23.12 ^b^	890.67 ± 34.04 ^a^	910.00 ± 41.79 ^a^	0.080
FCR, %	0.94 ± 0.01 ^a^	0.75 ± 0.02 ^b^	0.58 ± 0.01 ^c^	0.61 ± 0.02 ^c^	<0.001
ADG, g	20.59 ± 0.98 ^b^	20.30 ± 0.59 ^b^	22.84 ± 0.10 ^a^	23.33 ± 0.30 ^a^	<0.001
CF, g/cm^3^	0.79 ± 0.04 ^ab^	0.83 ± 0.01 ^a^	0.79 ± 0.04 ^ab^	0.75 ± 0.04 ^b^	0.876

IBW: initial body weight; FBW: final body weight; WG: weight gain; FCR: feed conversation ratio; ADG: average daily gain; CF: condition factor; The mean ± SEM was used to present the data. Values that had different superscripts within the same row exhibited significant differences (*p* < 0.05).

## Data Availability

For additional information or clarification, readers are encouraged to contact the authors directly. Supplementary data and relevant unpublished manuscripts can be provided upon request.
